# Impaired Cytokine Responses to Epstein-Barr Virus Antigens in Systemic Lupus Erythematosus Patients

**DOI:** 10.1155/2016/6473204

**Published:** 2016-03-27

**Authors:** Anette Holck Draborg, Noreen Sandhu, Nanna Larsen, Janni Lisander Larsen, Søren Jacobsen, Gunnar Houen

**Affiliations:** ^1^Department of Autoimmunology and Biomarkers, Statens Serum Institut, 2300 Copenhagen, Denmark; ^2^Department of Congenital Disorders, Statens Serum Institut, 2300 Copenhagen, Denmark; ^3^Department of Rheumatology, Rigshospitalet, Copenhagen University Hospital, 2100 Copenhagen, Denmark

## Abstract

We analyzed cytokine responses against latent and lytic Epstein-Barr virus (EBV) antigens in systemic lupus erythematosus (SLE) patients and healthy controls (HCs) to obtain an overview of the distinctive immune regulatory response in SLE patients and to expand the previously determined impaired EBV-directed T-cell response. The concentrations of 14 cytokines (IL2, IL4, IL5, IL6, IL10, IL12, IL17, IL18, IL1*β*, IFN*γ*, TNF*α*, TNF*β*, TGF*β*, and GM-CSF) were quantified upon stimulation of whole blood with latent state antigen EBNA1, lytic cycle antigen EBV-EA/D, and the superantigen SEB. To avoid results affected by lack of lymphocytes, we focused on SLE patients with normal levels. Decreased induction of IL12, IFN*γ*, IL17, and IL6 upon EBNA1 stimulation and that of IFN*γ*, IL6, TNF*β*, IL1*β*, and GM-CSF upon EBV-EA/D stimulation were detected in SLE patients compared to HCs. IFN*γ* responses, especially, were shown to be reduced. Induction of several cytokines was furthermore impaired in SLE patients upon SEB stimulation, but no difference was observed in basic levels. Results substantiate the previously proposed impaired regulation of the immune response against latent and lytic cycle EBV infection in SLE patients without lymphopenia. Furthermore, results indicate general dysfunction of leukocytes and their cytokine regulations in SLE patients.

## 1. Introduction

Systemic lupus erythematosus (SLE) is an autoimmune inflammatory disease that occurs mostly in women in the reproductive age. The typical disease course involves periods of disease flares alternating with remissions. The pathogenesis of SLE is complex and involves both genetic predispositions and environmental factors including viral infections [1–5].

Epstein-Barr virus (EBV) is especially associated with SLE and numerous studies have suggested a link including abnormally elevated viral load [6–8], pronounced EBV-directed antibody responses [9–22], and deficient EBV-specific T-cell responses [6, 23–25] in SLE patients compared to healthy controls (HCs). Nearly all SLE patients (99.5%) and also HCs (94.5%) are infected with EBV [15]. However, according to the many previous studies, SLE patients seem to experience reduced control of the latent infection.

Primary EBV infection is mostly asymptomatic or with mild symptoms during childhood but can cause infectious mononucleosis in adolescence [26, 27]. Subsequent to primary infection a latent state is established and the virus stays within memory B-cells and expresses only a limited set of genes including EBV nuclear antigen (EBNA) 1 [28–30]. The latent state is occasionally superseded by EBV reactivation and introduction into a lytic active cycle involving viral genome replication and viral gene expression [30, 31]. In immune-competent individuals, the immune response is able to control the EBV infection, and cell-mediated immunity is especially essential with interferon (IFN) *γ* suggested as a central mediator [32–34].

An imbalance of helper T-cells (Th) and variations in the complex and susceptible cytokine network [35, 36] play an important role in the pathogenesis of SLE. Studies on upregulated basic levels of especially IFN*α*, tumor necrosis factor (TNF) *α*, interleukin (IL) 6, IL10, and IL17 are proposed to be relevant in SLE [35]. Common gene variants involved in the pathogenesis of SLE moreover comprise components of various cytokine pathways including* IRF5*,* STAT4*,* IL6*, and* TNFSF4* [37, 38], which possibly could create disorders in the balance and regulation of cytokine responses upon antigen stimulation.

Cytokines affect multiple processes and function in a network of interacting cells with a specific interplay that is finely regulated and cytokines often hold contradictory functions according to the local environment.  Table 1 shows an outline of effector molecules examined in this study.

We have previously demonstrated a limited or defective T-cell response against EBV antigens in SLE patients, showing a reduced number of T-cells becoming activated and producing IFN*γ* upon EBV stimulation (both the latent antigen EBNA1 and the lytic cycle antigen EBV early antigen diffuse (EBV-EA/D)) [25]. Furthermore, Larsen et al. [39] revealed that fewer EBV-specific cytotoxic T lymphocytes (CTLs) are able to secrete IFN*γ*, TNF*α*, and IL2.

In order to examine the reduced T-cell response observed in SLE patients further and to obtain an overview of the distinctive immune regulatory response in SLE patients, the cytokine profile upon EBV antigen stimulation was determined in this study. With the purpose of investigating the response to both latent and lytic states of the EBV life cycle, stimulations with EBNA1 and EBV-EA/D were conducted. Furthermore, the cytokine responses' association with disease activity was examined.

## 2. Materials and Methods

### 2.1. SLE Patients and HCs

Whole blood samples from 27 SLE patients and 27 sex- and age-matched HCs were included in the current study. The age-matching was, for most SLE patient/HC pairs, +/− five years of age. However, for six SLE patient/HC pairs the HCs were 15–32 years younger than their matched SLE patients. The samples have previously been studied [25]. SLE patients were collected at Department of Infectious Diseases and Rheumatology, Rigshospitalet, Copenhagen, Denmark; all fulfilled internationally accepted classification criteria for SLE [40]. Blood samples from apparently HCs were from volunteers at Statens Serum Institut, Copenhagen, Denmark. Written informed consent for the studies was obtained from all patients according to the protocol approved by the Scientific Ethical Committee of the Capital Region of Denmark (number H-A-2007-0114).

Clinical characteristics of the included 27 SLE patients and 27 sex- and age-matched HCs are outlined in Table 2, left column. Furthermore, characteristics of the part of SLE patients (and corresponding HCs) with lymphopenia (<1.00 *∗* 10^9^/L) (*n* = 12) (middle column) and the part with normal lymphocyte levels (all also had normal leukocyte levels) (*n* = 15) (right column), respectively, are outlined. All symptoms, serological data, and information on medication in Table 2 are of time of blood collection for the current project.

Mean SLE disease activity index (SLEDAI) scores for all SLE patients was 6.0 (ranging from 0 to 22) and 5.5 (ranging from 0 to 15) and 6.7 (ranging from 0 to 22) for the part of SLE patients with lymphopenia and with normal lymphocyte levels, respectively.

### 2.2. *Ex Vivo* Stimulation of Whole Blood Samples with Viral Antigens

In parallel with previous* ex vivo* stimulation experiments utilized for intracellular cytokine measurements [25], the heparinized whole blood samples from SLE patients and HCs were stimulated for 24 hours at 37°C for subsequent measurements of secreted cytokines. Samples were stimulated with EBNA1 (5 *μ*g/mL,* Escherichia coli*-derived, EBV-271, Prospec Protein Specialist, Ness-Ziona, Israel) and EBV-EA/D (5 *μ*g/mL,* Escherichia coli*-derived, EBV-272, Prospec Protein Specialist, Ness-Ziona, Israel) and with staphylococcal enterotoxin B (SEB) (10 *μ*g/mL, Sigma-Aldrich, St. Louis Missouri, USA); phosphate-buffered saline (PBS) and* Escherichia coli*-derived *β*-galactosidase (5 *μ*g/mL, Sigma-Aldrich, St. Louis Missouri, USA) were prepared in parallel as positive and negative controls, respectively. The negative control with PBS stimulation was used to determine baseline levels and the negative control with *β*-galactosidase was used to ensure that the stimulation of cytokines was not due to the fact that the EBV antigens were produced in* Escherichia coli*. Following stimulation, cold 20 mM EDTA (100 *μ*L/mL) was added to the whole blood samples to stop the reaction and incubated for 15 minutes at room temperature. 100 *μ*L stimulated whole blood was stored at −20°C as dried blood spot samples (DBSS) on filter paper until all patients and HCs were included in the study. To facilitate comparability between SLE patients and HCs each sex- and age-matched SLE patient/HC pair was always stimulated and analyzed simultaneously and had the same lag time (time period from blood collection to beginning of stimulation, ranging from 44 to 133 minutes).

### 2.3. Multiplexed Luminex Assay

The concentrations of 14 cytokines secreted upon stimulation with EBNA1, EBV-EA/D, SEB, PBS, and *β*-galactosidase were analyzed with an in-house assay panel using the multiplex Luminex xMAP technology (Luminex Corp., TX, USA) as previously described in [41].

In short, two 3 mm disks were punched from each DBSS and extracted in 130 *μ*L buffer (phosphate-buffered saline (PBS) containing 0.5% Tween 20, 1% Bovine serum albumin (BSA), and one tablet of complete protease inhibitor cocktail (Roche, Germany) dissolved per 25 mL buffer) for 1 hour at RT. Then, 50 *μ*L of the extracted sample and 50 *μ*L of a suspension of capture antibody-conjugated beads were mixed in plate wells. After 1.5 hours of incubation, the beads were washed twice and subsequently reacted for 1.5 hours with a mixture (50 *μ*L) of corresponding biotinylated detection antibodies, each diluted 1 : 1000. Streptavidin-phycoerythrin (50 *μ*L) was added to the wells, and the incubation was continued for an additional 30 minutes. Finally, the beads were washed twice and resuspended in 125 *μ*L of buffer and analyzed on the Luminex 100*™* platform (Luminex Corp., TX, USA).

In addition to the standard assay conditions, heterophilic blocking reagent plus (HBR+) (3KC545 Scantibodies Laboratory, Inc., Santee, CA 92071, USA) was added both simultaneously to samples (both SLE and HC samples) and together with conjugate in a concentration of 400 *μ*g/mL to avoid false positive results due to rheumatoid factors. Preliminary experiments showed reliable blocking of rheumatoid factors by HBR+.

The working range (WR) for each analyte was defined as the concentration range in which the coefficient of variation (CV) was below 20%. Concentrations measured below the lowest concentrations in the working range were set as half the lowest concentration.

The WRs for the selected analytes were IL4, IL5, IL12, IL17, and IL1*β*: 8–4000 pg/mL; IL2, IL6, IL10, transforming growth factor (TGF) *β*, TNF*α*, and IFN*γ*: 156–80.000 pg/mL; IL18, TNF*β*, and granulocyte macrophage colony-stimulating factor (GM-CSF): 20–10.000 pg/mL.

Each Luminex plate contained a high and a low positive control together with a standard curve. The standard curves were fitted with a five-parameter logistic equation (Logistic-5PL) using BioPlex*™* Manager 6.1 (Bio-Rad Laboratories, CA, USA).

The plate setup comprised all samples from each sex- and age-matched SLE patient/HC pair on the same plate. In-house multiplex Luminex intra-assay variations and interassay variations are described in [41].

### 2.4. Statistical Analyses

Statistical analyses of data were carried out using GraphPad Prism Software 5 (GraphPad Software, Inc., La Jolla, California, USA). Comparisons of cytokine concentrations in sex- and age-matched SLE patients and HCs were performed using the two-sided Wilcoxon matched-pairs test, except for IL1*β* responses which were compared using the two-sided Chi-squared test (due to the fact that the results on IL1*β* responses were above the upper range of the assay for the majority of individuals). Data are presented as median with interquartile range with statistical significant differences indicated with *∗*, *∗∗*, or *∗∗∗* for *p* values below 0.05, 0.01, or 0.001, respectively. Univariate correlation analyses were performed using Spearman's correlation test for nonparametric data sets.

## 3. Results

In order to characterize the cytokine responses against EBV in SLE patients and compare them to HCs, 14 cytokines secreted upon stimulation with the latent state EBNA1 and the lytic cycle EBV-EA/D were quantified. To assess the issue without an effect on results due to lymphopenia (which is commonly observed in SLE patients), only SLE patients with normal lymphocyte levels (>1.00 *∗* 10^9^/L) were compared to their corresponding sex- and age-matched HCs in the following presented results. None of the selected SLE patients with normal lymphocyte levels experienced leukopenia.

No difference was observed between SLE patients and HCs in any of the 14 cytokine measurements upon PBS stimulation, which serves as basic levels of cytokines (results not shown).

Actually, a significant induced secretion of IL6 (median concentration of 8469 and 8349 pg/mL in SLE patients and HCs, resp.) and IL1*β* (median concentration of 2540 and 1301 pg/mL in SLE patients and HCs, resp.) was determined after stimulation with *β*-galactosidase which was used to determine the amount of cytokine stimulation originated from the* Escherichia coli* production system, thus making the results on these cytokines upon stimulation with EBNA1 and EBV-EA/D inconclusive. However, no significant differences in *β*-galactosidase-induced IL6 or IL1*β* were observed between SLE patients and HCs. Statistically significantly decreased secretion of IFN*γ*, IL12, GM-CSF, IL6, IL1*β*, and IL10 was observed in SLE patients compared to HCs upon SEB stimulation (Table 3). Due to the exclusion of SLE patients with lymphopenia, these results were not caused by lack of T-cells. However, these results could be a sign of dysfunctional leukocytes.

### 3.1. Cytokine Responses against the Latent State EBV Antigen EBNA1

**Figure 1:**
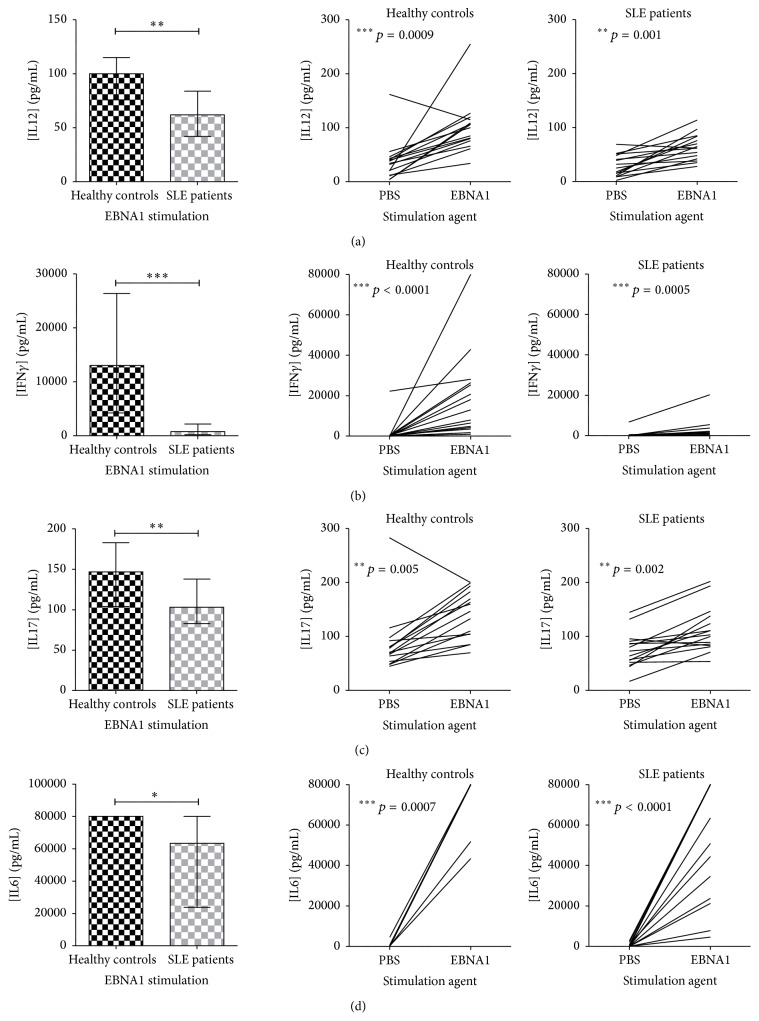
Decreased induction of IL12 (a), IFN*γ* (b), IL17 (c), and IL6 (d) in SLE patients compared to HCs upon EBNA1 stimulation. IL12 (a), IFN*γ* (b), IL17 (c), and IL6 (d) quantified in dried blood spot samples from SLE patients (with normal lymphocyte levels, *n* = 15) and sex- and age-matched HCs (*n* = 15) by multiplexed Luminex assay with addition of HBR+. Left: cytokine concentrations (pg/mL) in SLE patients and HCs upon EBNA1 stimulation. Data are presented as median with interquartile range. *p* values for comparison of SLE patients and HCs are 0.009, <0.0001, 0.003, and 0.027 in (a), (b), (c), and (d), respectively. Right: induction of cytokines (pg/mL) from basic levels (PBS stimulation) by EBNA1 stimulation in SLE patients and HCs.

The induction of IL12, IFN*γ*, IL17, and IL6 upon EBNA1 stimulation was statistically significantly decreased in SLE patients compared to HCs (Figure 1, left), even though they were all observed to be induced in both groups compared to PBS stimulation (Figure 1, right). The induction of IFN*γ* was especially impaired in SLE patients (Figure 1(b)). The difference between SLE patients and HCs upon EBNA1 stimulation remained significant even with the exclusion of a HC with high concentration of IL12 (255 pg/mL) and IFN*γ* (>80.000 pg/mL), respectively. IL6 was significantly more induced by EBNA1 (median concentration of 63449 and 80000 pg/mL in SLE patients and HCs, resp.) than with *β*-galactosidase (median concentration of 8469 and 8349 pg/mL in SLE patients and HCs, resp.) with *p* values of 0.0001 and <0.0001 for SLE patients and HCs, respectively.

**Figure 2:**
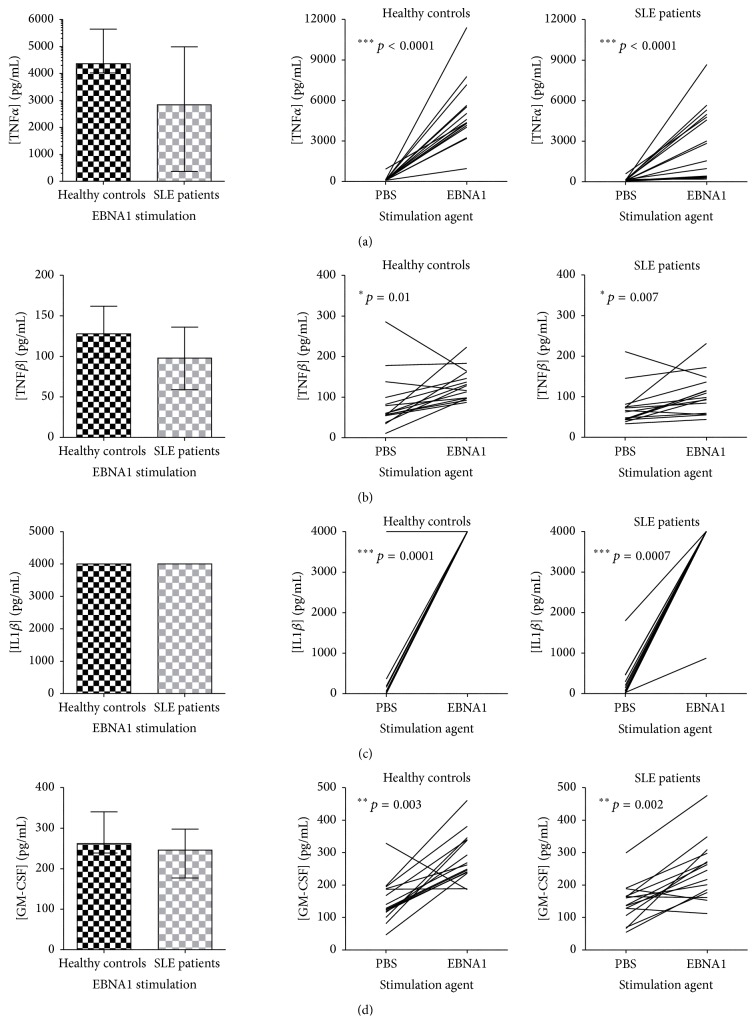
Inflammatory cytokines, TNF*α* (a), TNF*β* (b), IL1*β* (c), and GM-CSF (d), with similar induction in SLE patients and in HCs upon EBNA1 stimulation. TNF*α* (a), TNF*β* (b), IL1*β* (c), and GM-CSF (d) quantified in dried blood spot samples from SLE patients (with normal lymphocyte levels, *n* = 15) and sex- and age-matched HCs (*n* = 15) by multiplexed Luminex assay with addition of HBR+. Left: cytokine concentrations (pg/mL) in SLE patients and HCs upon EBNA1 stimulation. Data are presented as median with interquartile range. *p* values for comparison of SLE patients and HCs are 0.083, 0.103, 0.310, and 0.050 in (a), (b), (c), and (d), respectively. Right: induction of cytokines (pg/mL) from basic levels (PBS stimulation) by EBNA1 stimulation in SLE patients and HCs.

**Figure 3:**
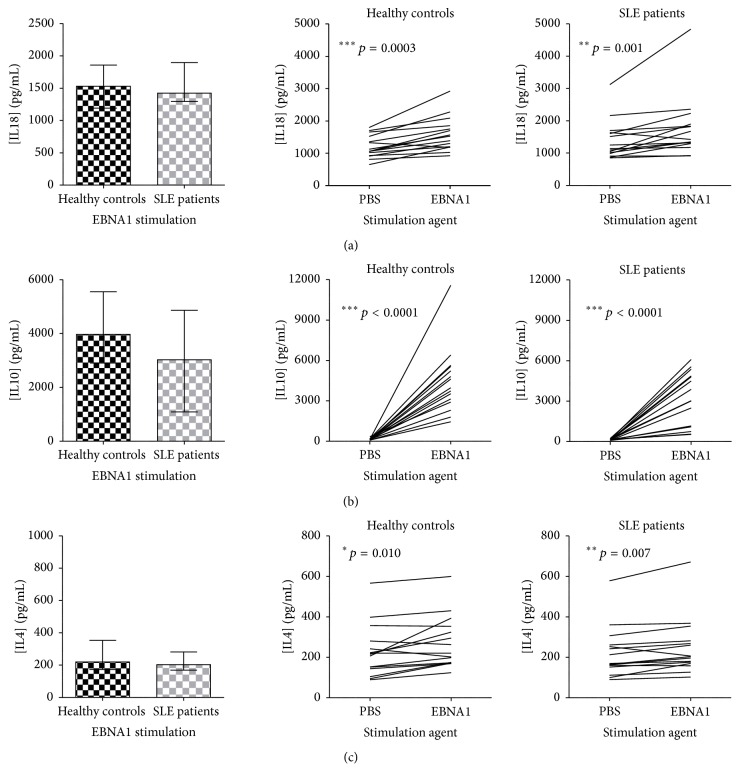
Cytokines involved in various T-cell responses, IL18 (a), IL10 (b), and IL4 (c), with similar induction in SLE patients and in HCs upon EBNA1 stimulation. IL18 (a), IL10 (b), and IL4 (c) quantified in dried blood spot samples from SLE patients (with normal lymphocyte levels, *n* = 15) and sex- and age-matched HCs (*n* = 15) by multiplexed Luminex assay with addition of HBR+. Left: cytokine concentrations (pg/mL) in SLE patients and HCs upon EBNA1 stimulation. Data are presented as median with interquartile range. *p* values for comparison of SLE patients and HCs are 0.762, 0.169, and 0.118 in (a), (b), and (c), respectively. Right: induction of cytokines (pg/mL) from basic levels (PBS stimulation) by EBNA1 stimulation in SLE patients and HCs.

The induction of the inflammatory cytokines TNF*α*, TNF*β*, IL1*β*, and GM-CSF (Figure 2) and also IL18, IL10, and IL4 (Figure 3), which are involved in various T-cell responses (IL18 and IL10 in Th1 response and IL4 in Th2 response), were all found to be normally induced in SLE patients subsequent to EBNA1 stimulation (Figure 2, left and Figure 3, left). Both SLE patients and HCs had a significant rise in concentrations of these cytokines upon stimulation with EBNA1 compared to PBS (Figure 2, right and Figure 3, right). IL1*β* concentrations were for all individuals (except one) above the upper range of the assay subsequent to EBNA1 stimulation making the results inconclusive. Compared to IL1*β*-induction upon *β*-galactosidase stimulation (median concentration of 1301 and 2540 pg/mL in SLE patients and HCs, resp.) the response to EBNA1 was higher in both SLE patients and HCs (median concentration of 4000 pg/mL for both) (*p* values of 0.0001 and 0.004 for SLE patients and HCs, resp.).

IL2, IL5, and TGF*β* were not induced upon stimulation with EBNA1 compared to PBS stimulation in either HCs or SLE patients (results not shown).

Spearman's test showed no direct correlation between the measured cytokine responses to EBNA1 in SLE patients and intake of immunosuppressant medication (results not shown).

### 3.2. Cytokine Responses against the Lytic Cycle EBV Antigen EBV-EA/D

**Figure 4:**
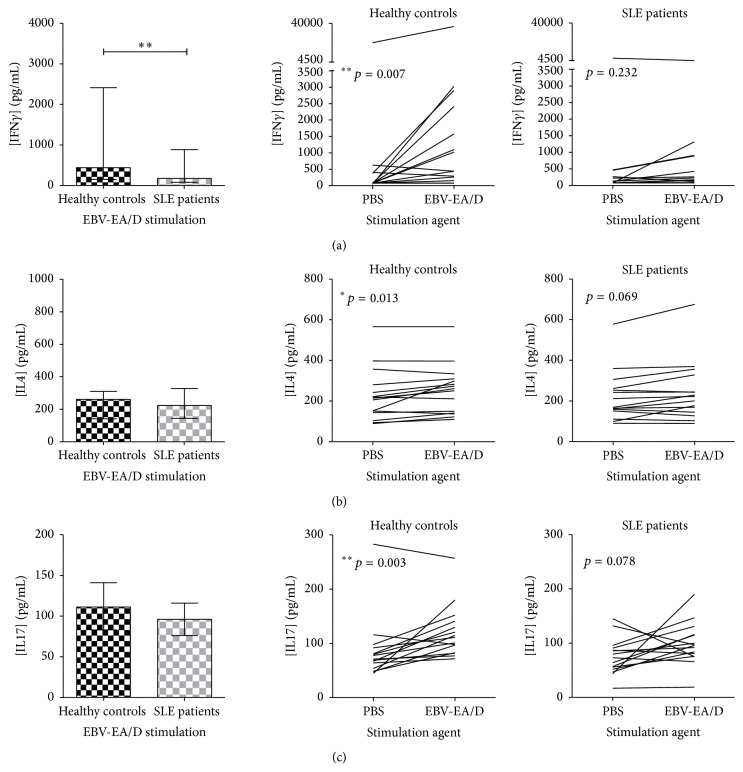
Impaired induction of IFN*γ* (a), IL4 (b), and IL17 (c) in SLE patients upon EBV-EA/D stimulation. IFN*γ* (a), IL4 (b), and IL17 (c) quantified in dried blood spot samples from SLE patients (with normal lymphocyte levels, *n* = 15) and sex- and age-matched HCs (*n* = 15) by multiplexed Luminex assay with addition of HBR+. Left: cytokine concentrations (pg/mL) in SLE patients and HCs upon EBV-EA/D stimulation. Data are presented as median with interquartile range. *p* values for comparison of SLE patients and HCs are 0.005, 0.443, and 0.191 in (a), (b), and (c), respectively. Right: induction of cytokines (pg/mL) from basic levels (PBS stimulation) by EBV-EA/D stimulation in SLE patients and HCs.

IFN*γ*, IL4, and IL17 concentrations were not induced in SLE patients upon EBV-EA/D stimulation, which was observed in HCs (Figure 4, right). This lack of induction of IFN*γ* in SLE patients resulted in a statistically significant difference between SLE patients and HCs in the concentration of IFN*γ* subsequent to EBV-EA/D stimulation (Figure 4(a), left). Yet, no significant difference was observed between SLE patients and HCs in the concentration of IL4 and IL17 subsequent to EBV-EA/D stimulation making the significant induction of these cytokines in HCs negligible (Figures 4(b) and 4(c)).

**Figure 5:**
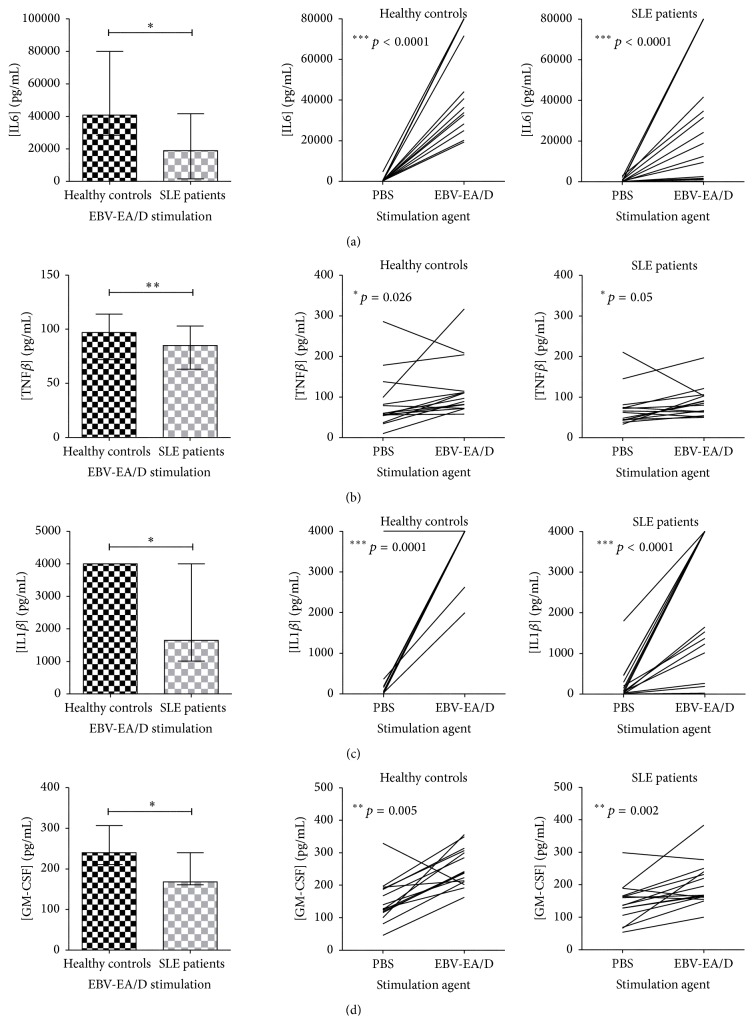
Decreased induction of IL6 (a), TNF*β* (b), IL1*β* (c), and GM-CSF (d) in SLE patients compared to HCs upon EBV-EA/D stimulation. IL6 (a), TNF*β* (b), IL1*β* (c), and GM-CSF (d) quantified in dried blood spot samples from SLE patients (with normal lymphocyte levels, *n* = 15) and sex- and age-matched HCs (*n* = 15) by multiplexed Luminex assay with addition of HBR+. Left: cytokine concentrations (pg/mL) in SLE patients and HCs upon EBV-EA/D stimulation. Data are presented as median with interquartile range. *p* values for comparison of SLE patients and HCs are 0.033, 0.010, 0.020, and 0.018 in (a), (b), (c), and (d), respectively. Right: induction of cytokines (pg/mL) from basic levels (PBS stimulation) by EBV-EA/D stimulation in SLE patients and HCs.

A statistically significantly decreased induction of IL6, TNF*β*, IL1*β*, and GM-CSF was observed in SLE patients compared to HCs upon EBV-EA/D stimulation (Figure 5, left). Yet, the cytokines were induced in both groups compared to PBS stimulation (Figure 5, right). Actually, IL1*β* concentrations subsequent to EBV-EA/D stimulation were for 13 of 15 HCs and for 7 of 15 SLE patients above the upper range of the assay making results inexact and a larger difference between SLE patients and HCs seems probable. However, compared to *β*-galactosidase-induced IL1*β* (median concentration of 1301 and 2540 pg/mL in SLE patients and HCs, resp.) the response to EBV-EA/D was higher in especially HCs (median concentration of 1650 and 4000 pg/mL in SLE patients and HCs, resp.) with a statistically significant difference for both SLE patients and HCs (*p* = 0.004 and 0.004, resp.). IL6-induction upon stimulation with EBV-EA/D (median concentration of 18884 and 40744 pg/mL in SLE patients and HCs, resp.) was significantly higher than with *β*-galactosidase (median concentration of 8469 and 8349 pg/mL in SLE patients and HCs, resp.) (*p* = 0.0004 and 0.0001 for SLE patients and HCs, resp.). Regarding TNF*β* and GM-CSF, a few individuals experienced a decrease in cytokine excretion upon EBV-EA/D compared to PBS stimulation.

**Figure 6:**
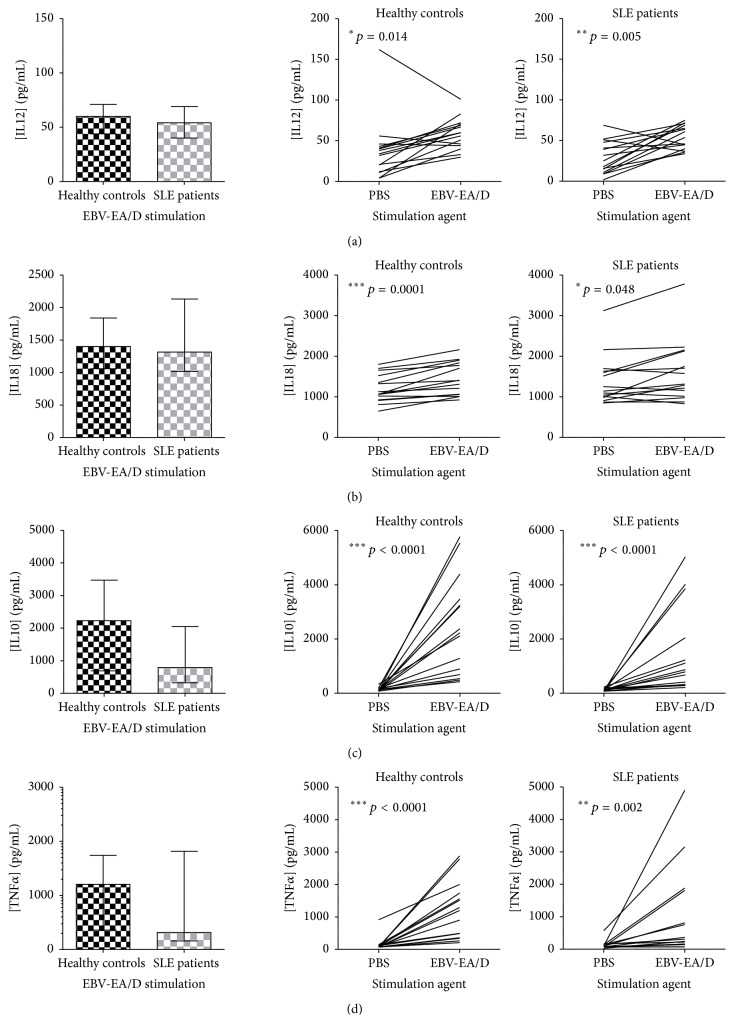
Cytokines, IL12 (a), IL18 (b), IL10 (c), and TNF*α* (d), with similar induction in SLE patients and in HCs upon EBV-EA/D stimulation. IL12 (a), IL18 (b), IL10 (c), and TNF*α* (d) quantified in dried blood spot samples from SLE patients (with normal lymphocyte levels, *n* = 15) and sex- and age-matched HCs (*n* = 15) by multiplexed Luminex assay with addition of HBR+. Left: cytokine concentrations (pg/mL) in SLE patients and HCs upon EBV-EA/D stimulation. Data are presented as median with interquartile range. *p* values for comparison of SLE patients and HCs are 0.639, 0.600, 0.107, and 0.600 in (a), (b), (c), and (d), respectively. Right: induction of cytokines (pg/mL) from basic levels (PBS stimulation) by EBV-EA/D stimulation in SLE patients and HCs.

IL12, IL18, and IL10 (all involved in regulation of the cell-mediated immune response) and the inflammatory cytokine TNF*α* were all similarly induced in SLE patients and HCs subsequent to EBV-EA/D stimulation compared to PBS stimulation (Figure 6, right) and with no difference in concentrations after stimulation (Figure 6, left).

As with EBNA1 stimulation, IL2, IL5, and TGF*β* were not induced upon stimulation with EBV-EA/D compared to PBS stimulation in either HCs or SLE patients (results not shown).

Spearman's test showed no direct correlation between the measured cytokine responses to EBV-EA/D in SLE patients and intake of immunosuppressant medication (results not shown).

### 3.3. Cytokine Responses and Disease Activity of SLE Patients

As shown in Table 4 neither SLEDAI scores nor clinical SLEDAI scores (cSLEDAI, i.e., SLEDAI score excluding anti-dsDNA, complement, thrombocyte, and leukocyte levels) correlated with cytokine responses in SLE patients. However serologic SLEDAI scores (sSLEDAI, i.e., SLEDAI score based on anti-dsDNA, complement, thrombocyte, and leukocyte levels) correlated negatively with numerous cytokine responses against EBNA1 and EBV-EA/D. IFN*γ*, IL4, TNF*α*, and TNF*β* responses to EBNA1 and IL18, IL4, and IL6 responses to EBV-EA/D correlated negatively with sSLEDAI scores of SLE patients, respectively. IL2, IL5, and TGF*β* results were not included in the correlation-study, as these cytokines were not induced upon stimulation. IL1*β* results were not included since the majority of measurements were above the upper range of the assay.

### 3.4. Cytokine Responses to Epstein-Barr Virus Antigens in SLE Patients according to Lymphocyte Levels

12 of the included 27 SLE patients suffered from lymphopenia (defined as lymphocyte levels <1.00 *∗* 10^9^/L). In the preceding results, only SLE patients with normal lymphocyte levels were compared to their corresponding sex- and age-matched HCs (*n* = 15). In Table 5, the *p* values for comparisons of cytokine concentrations in SLE patients with HCs are stated comparing all SLE patients/all HCs (*n* = 27), only SLE patients with lymphopenia (and corresponding HCs) (*n* = 12), and also only SLE patients with normal lymphocyte levels (and corresponding HCs) (*n* = 15, illustrated in preceding results) in order to evaluate the influence of missing lymphocytes on cytokine production and release. The majority of cytokine responses were decreased in SLE patients disregarding lymphocyte levels (all SLE, *n* = 27). However, according to Table 5, many of these results were due to the SLE patients with lymphopenia (*n* = 12).

## 4. Discussion

In this study, the cytokine responses against EBNA1 (latent state) and EBV-EA/D (lytic cycle) were investigated in SLE patients and HCs by multiplexed Luminex technology. The focus was on SLE patients with normal lymphocyte levels in order to avoid results merely caused by a lack of T-cells. The quantified responses were decreased in SLE patients compared to HCs regarding numerous cytokines and, furthermore, secretion of some cytokines was not induced in SLE patients but in HCs when compared to PBS stimulation (served as basic cytokine levels). Quantified cytokines and lymphocyte levels of SLE patients did not correlate, suggesting that the lower cytokine responses observed in SLE patients were not due to lymphocyte levels in the lower region of the normal range (as all included SLE patients in this analysis have lymphocyte levels above the threshold) (results not shown).

No difference in basic levels (PBS stimulation) of any of the 14 quantified cytokines was observed between SLE patients and HCs indicating that the SLE patients without lymphopenia actually have a normal distribution of cytokines without an aberrant exaggerated immune stimulation. Importantly, these results ensure that the difference in cytokine responses upon EBV stimulation is not due to variations in basic levels. However, previous studies on basic levels of cytokines in SLE patients have shown increased levels of especially inflammatory cytokines [35], but these results were based on cohorts disregarding lymphocyte levels.

Stimulation with* Escherichia coli*-derived *β*-galactosidase induced the secretion of IL6 and IL1*β* in both SLE patients and HCs. As not all 14 cytokines were induced upon *β*-galactosidase this outcome was presumably not due to a general effect of lipopolysaccharide from the production in* Escherichia coli*. Furthermore, the stimulation with EBNA1 and EBV-EA/D (both* Escherichia coli*-derived) entailed significantly larger amounts of cytokine release than stimulation with *β*-galactosidase.

SLE patients exhibited a decreased IL12, IFN*γ*, IL17, and IL6 response to the latent state EBV antigen EBNA1. IFN*γ* and IL12 are both important factors in Th1 differentiation and are crucial in enhancement of both CTL activity and NK cell activity. IL12 stimulates IFN*γ* production from CTLs and NK cells and IFN*γ* furthermore increases macrophage activity. Accordingly, the current results could be a result of impaired Th1, Th17, CTL, and NK cell regulations and furthermore a decreased acute phase response against the latent state EBV infection in SLE patients (which is not a result of decreased number of lymphocytes). However, results on quantified TNF*β*, which is produced by Th1 cells in response to viral infection [49, 50], remarkably showed similar induced secretion in both HCs and SLE patients upon EBNA1 stimulation. Thus, not all cells in the EBNA1-specific T-cell subpopulation in SLE patients are deficient or dysfunctional. It could be hypothesized that a population of EBNA1-specific T-cells in SLE patients are functionally inhibited perhaps through costimulatory/coinhibitory pathway factors including CTLA4 and PD1 [39]. This would presumably result in dysregulation and weak responses to EBV as observed in the current study.

As IL1*β*, TNF*α*, IL18, and GM-CSF secretion was induced to a normal level upon EBNA1 stimulation in SLE patients, the macrophages of SLE patients are assumedly functional and responsive to the latent EBV infection. A similar impaired cytokine response was observed to the lytic cycle EBV antigen EBV-EA/D in SLE patients. IFN*γ*, IL4, and IL17 were not induced at all in SLE patients upon EBV-EA/D stimulation, yet a difference between SLE patients and HCs was only seen regarding IFN*γ*. Furthermore, the impaired cytokine response to lytic EBV infection involved IL6, TNF*β*, IL1*β*, and GM-CSF. Similar to the EBNA1-directed response, these results could be due to impaired Th1, (Th17), CTL, NK cell regulations, and acute phase responses against lytic EBV infection, besides a lack of the antiviral functions of TNF*β*, yet with reasonable functioning macrophages producing IL12, IL18, and TNF*α* perhaps aiming (unsuccessfully) to induce IFN*γ* and thereby cell-mediated immunity and also to activate NK cells and mediate inflammation.

Previous studies have shown increased titers of antibodies to EBV-EA/D [54]. Thus the impaired induction of IL4 in response to EBV-EA/D in SLE patients was actually unexpected.

Overall, results show especially impaired IFN*γ* responses in SLE patients against both latent and lytic EBV infection. Actually, current results on the cytokine profile upon EBV stimulation imply that, exclusively, IFN*γ* measurements would be sufficient in the monitoring of the EBV response. We have previously demonstrated a significantly decreased level of intracellular IFN*γ* in EBV-specific T-cells in the same cohort as investigated in this study [25]. Spearman's analyses demonstrated strong correlations between previously measured intracellular IFN*γ* and extracellular (secreted) IFN*γ* measured in this study after both EBNA1 and EBV-EA/D stimulation (*r* = 0.846 (*p* < 0.0001) and *r* = 0.470 (*p* = 0.004), resp.) validating the results. Current results on impaired Th1- and CTL-derived cytokines upon stimulation additionally confirm our previous results on limited or defective cell-mediated immunity to both latent (EBNA1) and lytic (EBV-EA/D) EBV infection. Furthermore, the low IFN*γ* response in SLE patients could also be due to an impaired NK cell response.

As also proposed by Larsen et al. [39] and in our previous work [25] the decreased cell-mediated response against EBV could be a result of frequent reactivations and thus hyperactivation and subsequent exhaustion of EBV-specific T-cells upon the continuous exposure to EBV. Another hypothesis is direct infection of T-cells with EBV [55–57] and subsequent destruction of the infected cells. It is speculated that, upon recurrent EBV reactivations in infected B-cells, EBV-specific T-cells would presumably be attracted to the site of EBV-producing B-cells and thus be an obvious target of infection by the newly synthesized EBV virions. Subsequently, new T-cells are recurrently produced (in SLE patients with normal levels of lymphocytes) but these will have random specificity (until selection) resulting in only a small pool of EBV-specific T-cells not being able to fight the persistent EBV infection. Consequently, this will entail poor control of the EBV infection which presumably results in a vicious cycle of recurrent reactivations and more widespread infection. Furthermore, reactivation and dissemination of the EBV infection presumably lead to enhanced expression of the lytic cycle viral IL10 homologue (vIL10) that (similar to human IL10) inhibits the synthesis of IFN*γ* and suppresses CTL activity and upregulation of MHC-I [58], which may contribute to the impaired cytokine response observed here. SLEDAI scores of SLE patients did not correlate with cytokine responses against EBNA1 or EBV-EA/D perhaps due to very low levels of several cytokines induced upon stimulation.

Only the serologic elements of SLEDAI (sSLEDAI, i.e., SLEDAI score based on anti-dsDNA, complement, thrombocyte, and leukocyte levels) and not the clinical elements (cSLEDAI, i.e., SLEDAI score excluding anti-dsDNA, complement, thrombocyte, and leukocyte levels) correlated with low cytokine responses in the SLE patients. Cytokine responses presumably reflect the regulations of both leukocytes and autoimmune humoral responses in the blood samples from SLE patients but not the clinical manifestations. Furthermore, serologic disease activity in the SLE patients indicates ongoing inflammation, which may be related to EBV reactivations due to impaired EBV-specific T-cell responses including the demonstrated correlations with low cytokine responses to EBV.

In addition to impaired cytokine responses to EBV antigens, the SLE patients also had a reduced secretion of IFN*γ*, IL12, GM-CSF, IL6, IL1*β*, and IL10 upon SEB stimulation compared to HCs, not caused by a lack of T-cells. Contrary to this study, the T-cells of SLE patients and HCs responded similarly upon SEB stimulation also in regard to intracellular IFN*γ* production in our previous work examining the same patients and HCs [25]. Results in this study could reflect dysfunctional leukocytes in general in SLE patients without lymphopenia. No direct correlation was found between the decreased measured cytokine responses and intake of immunosuppressant medication indicating that these results are not caused by an iatrogenically suppressed immune system in SLE patients. However, it cannot be ruled out that medications may have induced certain intrinsic immune defects including functional impairments of lymphocytes.

As anticipated, the SLE patients with lymphopenia experienced deficiencies in the majority of the EBV-induced cytokines compared to HCs, with the missing lymphocytes affecting the cytokine production and release. Accordingly, a larger percentile of SLE patients with lymphopenia received immunosuppressant medication but actually they did not have higher disease activities (mean SLEDAI of 5.5, range 0–15) than SLE patients with normal lymphocyte levels (mean SLEDAI of 6.7, range 0–22) suggesting that the lymphocyte levels are not a main factor in the disease manifestations.

In conclusion, results obtained in this study expand previously suggested impaired latent and lytic EBV-specific cell-mediated responses in SLE patients. In the demonstrated variance in the reflection of cytokines in response to latent and lytic EBV infection most cytokines originating from T-cells were impaired but with assumedly rather functioning macrophages responding to EBV. Yet not all cytokine responses primarily expressed by T-cells seemed to be impaired which could be due to subpopulations of repressed EBV-specific T-cells. In general, a poor regulation of the immune response against EBV in SLE patients is proposed.

## Figures and Tables

**Table 1:** Overview of cytokines included in this study.

Cytokine	Main origin	Central functions	References
IL2	T-cells	Growth and differentiation of T-cells NK (natural killer) cells activity	[42]

IL12	Macrophages, dendritic cells	Th1 differentiation NK cell activation Promoting IFN*γ*-secretion from T-cells and NK cells	[43]

IFN*γ*	Th1, CTL, NK cells	Differentiation and activation of T-cells, NK cells, macrophages Promotes IL12-secretion from macrophages and dendritic cells Increases antigen presentation via MHC expression Antiviral activity	[44]

IL18	Macrophages	Promotes IFN*γ* secretion from T-cells and NK cells	[45]

IL4	Th2, mast cells	Th2 differentiation B-cell activation and differentiation into antibody-secreting plasma cells	[46]

IL5	Th2, mast cells	B-cell growth and activity	[47]

TNF*α*	Macrophages	Inflammatory mediator	[48]

TNF*β*	Th1	Inflammatory mediator Enhance adhesion Antiviral activity	[49, 50]

IL6	Macrophages, Th2, B-cells	Both inflammatory and anti-inflammatory Involved in acute phase response	[35]

IL17	Th17	Inflammatory mediator Increases chemokine and cytokine production	[51]

IL1*β*	Macrophages	Inflammatory mediator	[52]

TGF*β*	Macrophages	Anti-inflammatory	[52]

IL10	CTL, Th, macrophages	Anti-inflammatory Downregulates cell-mediated immunity Negative feedback regulator	[35]

GM-CSF	T-cells, macrophages, endothelial cells	Inflammatory Stimulates production of granulocytes and monocytes from stem cells	[53]

IL: interleukin; IFN: interferon; TNF: tumor necrosis factor; GM-CSF: granulocyte macrophage colony-stimulating factor; CTL: cytotoxic T lymphocytes; NK: natural killer; Th: T helper cells.

**(a):** 

	All SLE patients	SLE patients with lymphopenia	SLE patients with normal lymphocyte levels
Number of individuals	27	12	15
Mean age (years) [range]	42.4 [21–81]	42.1 [21–81]	42.7 [21–72]
Females	96%	92%	100%
Disease manifestations:			
Nephritis	30%	25%	33%
Vasculitis	0%	0%	0%
Arthritis	26%	17%	33%
Rash	19%	17%	20%
Alopecia	0%	0%	0%
Myositis	0%	0%	0%
Mucosal ulcers	0%	0%	0%
Serositis	7%	0%	13%
Leukopenia	7%	17%	0%
Thrombocytopenia	22%	42%	7%
Visual disturbance	0%	0%	0%
Fever	11%	8%	13%
Mean SLEDAI [range]	6.0 [0–22]	5.5 [0–15]	6.7 [0–22]
dsDNA antibody positive	48%	42%	53%
Rheumatoid factor positive			
IgA	19%	17%	20%
IgM	4%	0%	7%
Low C3 or C4 level	52%	67%	40%
Mean C-reactive protein (mg/L) [range]	5 [1–22]	4 [1–15]	6 [1–22]
Medication:			
Prednisolone	59%	83%	40%
Azathioprine	26%	42%	13%
Mycophenolate mofetil	22%	17%	27%
Methotrexate	7%	8%	7%
Hydroxychloroquine	52%	67%	40%
Anticoagulant	33%	50%	20%
Antihypertension	26%	17%	40%
EBNA1 IgG antibodies	89%	92%	87%

**(b):** 

	All HCs	HCs matched to SLE patients with lymphopenia	HCs matched to SLE patients with normal lymphocyte levels
Number of individuals	27	12	15
Mean age (years) [range]	37.2 [22–61]	33.6 [23–59]	40.1 [22–61]
Females	93%	92%	93%
EBNA1 IgG antibodies	96%	92%	100%

SLE: systemic lupus erythematosus; SLEDAI: SLE disease activity index; ANA: nuclear antibodies; dsDNA: double stranded DNA; HCs: healthy controls.

**Table 3:** Cytokine responses in SLE patients and HCs upon SEB stimulation.

Cytokine	Median concentration (pg/mL) [range] in SLE patients (*n* = 15)	Median concentration (pg/mL) [range] in HCs (*n* = 15)	*p* value for comparison of SLE patients and HCs
IL2	1988 [78–6827]	2650 [78–4870]	0.169
IL12	64 [29–159]	97 [27–170]	0.035^*∗*^
IFN*γ*	20509 [1376–80000]	67048 [16193–80000]	0.001^*∗∗∗*^
IL18	1240 [818–2625]	1126 [773–2035]	0.359
IL4	281 [111–1202]	362 [139–678]	0.107
IL5	13 [4–138]	36 [13–116]	0.268
TNF*α*	891 [78–3941]	2002 [625–4572]	0.083
TNF*β*	344 [25–1242]	649 [244–1172]	0.083
IL6	3476 [78–20521]	9141 [4340–33403]	0.026^*∗*^
IL17	433 [57–2599]	669 [259–4000]	0.064
IL1*β*	4000 [93–4000]	4000 [4000-4000]	0.012^*∗*^
TGF*β*	1450 [568–5485]	1490 [643–6211]	0.121
IL10	811 [160–2276]	1920 [444–4491]	0.003^*∗∗*^
GM-CSF	475 [112–1620]	1236 [554–3713]	0.005^*∗∗*^

SEB: staphylococcal enterotoxin B; SLE: systemic lupus erythematosus; HCs: healthy controls; IL: interleukin; IFN: interferon; TNF: tumor necrosis factor; GM-CSF: granulocyte macrophage colony-stimulating factor.

All cytokine responses were compared using Wilcoxon matched-pairs test, except for IL1*β* responses, which were compared using the Chi-squared test.

**Table 4:** Correlation between induced cytokines and disease activity of SLE patients (*n* = 15).

Cytokine	SLEDAI	cSLEDAI	sSLEDAI
(A) EBNA1 stimulation			
IL12	NS	NS	NS
IFN*γ*	NS	NS	−0.757 (0.001^*∗∗*^)
IL18	NS	NS	NS
IL4	NS	NS	−0.747 (0.001^*∗∗*^)
TNF*α*	NS	NS	−0.619 (0.014^*∗*^)
TNF*β*	NS	NS	−0.548 (0.034^*∗*^)
IL6	NS	NS	NS
IL17	NS	NS	NS
IL10	NS	NS	NS
GM-CSF	NS	NS	NS

(B) EBV-EA/D stimulation			
IL12	NS	NS	NS
IFN*γ*	NS	NS	NS
IL18	NS	NS	−0.610 (0.016^*∗*^)
IL4	NS	NS	−0.595 (0.019^*∗*^)
TNF*α*	NS	NS	NS
TNF*β*	NS	NS	NS
IL6	NS	NS	−0.625 (0.013^*∗*^)
IL17	NS	NS	NS
IL10	NS	NS	−0.633 (0.011^*∗*^)
GM-CSF	NS	NS	NS

SLE: systemic lupus erythematosus; SLEDAI: SLE disease activity index; cSLEDAI: clinical SLEDAI; sSLEDAI: serologic SLEDAI; EBNA: Epstein-Barr virus (EBV) nuclear antigen; EBV-EA/D: Epstein-Barr virus early antigen diffuse; IL: interleukin; IFN: interferon; TNF: tumor necrosis factor; GM-CSF: granulocyte macrophage colony-stimulating factor; NS: not significant.

All values are spearman *r* (*p* values).

SLEDAI: Systemic Lupus Erythematosus Disease Activity Index score.

cSLEDAI: SLEDAI score excluding anti-dsDNA, complement, thrombocyte, and leukocyte levels.

sSLEDAI: SLEDAI scores based on anti-dsDNA, complement, thrombocyte, and leukocyte levels.

NS: not significant.

**Table 5:** *p* values for comparison of cytokine concentrations in SLE patients and HCs®.

Cytokine	All SLE and corresponding HCs (*n* = 27)	SLE with normal lymphocyte levels and corresponding HCs (*n* = 15)	SLE with lymphopenia and corresponding HCs (*n* = 12)
EBNA1 stimulation			
IL2	1.000	1.000	1.000
IL12	<0.0001^*∗∗∗*^	0.009^*∗∗*^	0.002^*∗∗*^
IFN*γ*	<0.0001^*∗∗∗*^	<0.0001^*∗∗∗*^	0.0005^*∗∗∗*^
IL18	0.095	0.762	0.233
IL4	0.002^*∗∗*^	0.118	0.005^*∗∗*^
IL5	0.038^*∗*^	0.232	0.092
TNF*α*	0.0004^*∗∗∗*^	0.083	0.0005^*∗∗∗*^
TNF*β*	0.0006^*∗∗∗*^	0.103	0.001^*∗∗*^
IL6	0.002^*∗∗*^	0.027^*∗*^	0.037^*∗*^
IL17	0.0001^*∗∗∗*^	0.003^*∗∗*^	0.014^*∗*^
IL1*β*	0.083	0.310	0.059
TGF*β*	0.008^*∗∗*^	0.188	0.027^*∗*^
IL10	0.008^*∗∗*^	0.169	0.012^*∗*^
GM-CSF	0.0002^*∗∗∗*^	0.05	0.003^*∗∗*^

EBV-EA/D stimulation			
IL2	0.500	0.500	1.000
IL12	0.361	0.639	0.480
IFN*γ*	0.0001^*∗∗∗*^	0.005^*∗∗*^	0.001^*∗∗∗*^
IL18	0.023^*∗*^ (SLE↑)	0.510	0.007^*∗∗*^ (SLE↑)
IL4	0.038^*∗*^	0.443	0.045^*∗*^
IL5	0.083	0.191	0.262
TNF*α*	0.035^*∗*^	0.510	0.016^*∗*^
TNF*β*	0.053	0.010^*∗∗*^	0.969
IL6	0.0006^*∗∗∗*^	0.033^*∗*^	0.001^*∗∗∗*^
IL17	0.044^*∗*^	0.191	0.129
IL1*β*	0.021^*∗*^	0.020^*∗*^	0.133
TGF*β*	0.0774	0.847	0.0210^*∗*^
IL10	0.0012^*∗∗*^	0.107	0.0015^*∗∗*^
GM-CSF	0.0006^*∗∗∗*^	0.018^*∗*^	0.0068^*∗∗*^

SLE: systemic lupus erythematosus; HCs: healthy controls; IL: interleukin; IFN: interferon; TNF: tumor necrosis factor; GM-CSF: granulocyte macrophage colony-stimulating factor; EBNA: Epstein-Barr virus (EBV) nuclear antigen; EBV-EA/D: Epstein-Barr virus early antigen diffuse.

®Significant differences between SLE patients and HCs in cytokine measurements correspond to highest values in HCs unless otherwise stated.

All cytokine responses were compared using the Wilcoxon matched-pairs test, except for IL1*β* responses, which were compared using the Chi-squared test.
